# Determinants of Termite Assemblages’ Characteristics within Natural Habitats of a Sudano-Guinean Savanna (Comoe National Park, Côte d’Ivoire)

**DOI:** 10.3390/insects9040189

**Published:** 2018-12-10

**Authors:** N’golo Abdoulaye Koné, Kolotchèlèma Simon Silué, Souleymane Konaté, Karl Eduard Linsenmair

**Affiliations:** 1 UFR des Sciences de la Nature (UFR SN), Unité de Recherche en Ecologie et Biodiversité (UREB), Université Nangui Abrogoua, 02 BP 801 Abidjan 02, Ivory Coast; silue.simon04@gmail.com (K.S.S.); konatesoul.sn@univ-na.ci (S.K.); 2 Station de Recherche en Ecologie du Parc National de la Comoé, 27 BP 847 Abidjan 27, Ivory Coast; ke_lins@biozentrum.uni-wuerzburg.de; 3 Tierökologie und Tropenbiologie (Zoologie III), Animal Ecology and Tropical Biology, Am Hubland, Julius-Maximilians-Universität Würzburg, D-97074 Würzburg, Germany

**Keywords:** termite assemblages, environnemental variables, Sudano-Guinean savanna, Comoe National Park, Côte d’Ivoire

## Abstract

Termites are one of the major components of tropical ecosystems. However, the ecological and biological variables determining the structure of their communities within natural habitats are less documented in general and especially in the Comoe National Park, a Sudano-Guinean savanna zone located in the north-eastern part of Côte d’Ivoire (West Africa). Using a standardized method of belt transects, the structure of termite’s communities was estimated within habitats differing in the structure of their vegetation, soil characteristics, and the disturbance level caused by annual occurrences of bushfires. The effect of a set of environmental variables (habitat type, occurrence of annual bushfire, woody plant density, woody plant species richness, and soil physicochemical parameters) was tested on the habitat-specific recorded termite species. Sixty species of termites belonging to 19 genera, seven subfamilies and two families, namely Rhinotermitidae (Coptotermitinae and Rhinotermitinae) and Termitidae (Apicotermitinae, Cubitermitinae, Macrotermitinae, Nasutitermitinae, and Termitinae) were sampled. These species were assigned to the four feeding groups of termites: fungus growers (18 species), wood feeders (17 species), soil feeders (19 species) and the grass feeders (6 species). The highest diversity of termites was encountered in forest habitats, with 37 and 34, respectively, for the gallery forest and the forest island. Among savanna habitats, the woodland savanna was identified as the most diversified habitat with 32 recorded species, followed by the tree savanna (28 species) and the grassy savanna (17 species). The distribution of termite species and their respective feedings groups was determined by the habitat type and a set of environmental variables such as Woody Plant Diversity (WPD), Woody plant Families Diversity (WPFD), and Organic Carbon (OC). The annual Fire Occurrence (FO) was found to indirectly impact the characteristics of termite assemblages within natural habitats via their respective Herbaceous Species Richness (HSR) and Woody Plant Species Richness (WPSR). In summary, the spatial heterogeneity of the Comoe National Park, modeled by the uncontrolled annual bushfire, offers a diversified natural habitat with an important variety of termite-habitat-specific species, probably due to the food preference of these organisms and its relatively good conservation status.

## 1. Introduction

Termites are one of the components that are actors and driving forces in the functioning and dynamics of tropical ecosystems [1,2,3], in addition to being the most dominant invertebrates of the soils of these ecosystems. These organisms represent more than 10% of animal kingdom biomass and around 95% of the total soil insect biomass [4]. Termites are of great importance with regard to the structuring and dynamics of tropical ecosystems and their functioning [5]. They improve soil ventilation, enhance absorption and storage of water in soils, and facilitate carbon storage [6,7]. In tropical dry forests, they consume between 40% to 100% of the dead wood, and in savannas up to 20% of the grass and leaf litters [8]. These organisms are also known as good bio-indicators in tropical ecosystems with regard to their sensitivity to habitat disturbance causing changes in their species richness, composition, and functional characteristics [9,10,11]. In these tropical ecosystems, such as savannas, termites’ community composition is strongly influenced by a set of parameters, like the variation in topography and soil characteristics [12,13], while changes in savanna vegetation structure and composition are mainly determined by precipitation, fire, and herbivory [14].

Termites are particularly abundant in savanna [15] and represent a keystone group of the ecology of these ecosystems [3]. In the moist savanna of the Lamto Reserve in Central Côte d’Ivoire, where fire was identified as the most important and frequently employed management tool for the maintenance of the savannas, different patterns of habitat-specific species richness was observed with regard to the fire-history of habitats [16]. In the Comoe National Park, a Sudano-Guinean savanna in the north-eastern part of the country, despite the unofficial use of fire as a management tool, the park is also burnt every year. Very little is known about the consequences of such an uncontrolled fire-induced habitat variability on the termite diversity. Furthermore, (i) determinants of the diversity and distribution of assemblages of these organisms within ecosystems remain understudied; yet they are essential for the biomonitoring of the protected area and (ii) the exact parameters governing community composition and distribution within such a heterogeneous landscape of savannas remain poorly understood.

The prime goal of this study was to quantify trends in diversity, spatial distribution, and abundance of termite’s communities of a Sudano-Guinean savanna and improve our understanding of their dynamics by identifying the major drivers of change, whether strictly environmental or resulting from biological factors. Specifically, it aimed at (i) estimating termite’s diversity and abundance in a Sudano-Guinean savanna, (ii) comparing characteristics of termite communities within the different habitat types of this savanna zone, and (iii) determining the biological and environmental factors governing such characteristics within these habitats. The diversity and abundance of termite assemblages were assessed across habitats of a Sudano-Guinean savanna (Comoe National Park, north-eastern part of Côte d’Ivoire in West Africa), differing in their (1) vegetation cover, (2) woody plant density, (3) plant species richness, (4) soil physicochemical parameters, and (5) the occurrence or disturbance level of annual bushfire. This kind of assessment among a gradient of habitat types would help in understanding various aspects of the savanna system processes and make the use of termites as biologic indicators of habitat disturbance easier.

## 2. Material and Methods

### 2.1. Study Site and Visited Habitats

The Comoe National Park (CNP) is a UNESCO World Heritage site and Biosphere Reserve located in North-Eastern Côte d’Ivoire (8°30′–9°40′ N and 3°10′–4°20′ W). This park of 11,500 km^2^, is one of the biggest protected areas in West Africa [17]. The mean annual precipitation recorded until the 1960s was 1150 mm [18] while the mean annual temperature was 27 °C. Rainfall occurs during one rainy season, from March to October while the dry season is observed from November to February. The rainiest month is September and the warmest month is January. The mean annual relative air humidity is 60% [19] and can reach 90% during the rainy season.

The park is located in a part of the Côte d’Ivoire characterized by a smooth and level relief. Soils are impoverished sandy to loam foresails above Precambrian granites with small areas of lateritic crust outcrops at certain places [20]. 

Due to the presence of the Comoe River, this park is an area of transitional habitats ranging from forests to savannas which include all types of savanna, forests, and riparian grasslands. The CNP is a semi-natural mosaic of forest–savanna with the main anthropogenic impact being uncontrolled annual fire by poachers. Savanna ecosystems cover 84.2% of this park. Gallery forests are found along the River Comoe and its tributaries with an estimated coverage of 2.3%. Islands of dry forests cover 8.4% while a particular type of grassland growing on ferralitic soils covers 4.9% [20,21].

This study was carried out in the southern part of the CNP, where the forest species of the Northern Guinean zone are gradually replaced by savanna species of the Sudanian zone towards the north. Five habitat types differing in the structure of their vegetation and the disturbance level caused by the annual bushfire were visited, characterized, and sampled:

A gallery forest (GF; 8°46′26.2″ N and 3°46′53.5″ W): located along the Comoe River, is characterized by a high tree density with a very closed canopy (2–5 large trees/100 m^2^). A significant quantity of leaf litter and fallen branches were observed on the soil. This habitat is in close proximity to savanna habitats but is naturally protected from the annual bushfire. Sixteen woody plants species were recorded in this habitat. Dominant plant species found in this habitat mainly belong to seven plant families: Euphorbiacece (*Drypetes floribunda*, *Dichapetalum madagascariensis*, *Drypetes gilgiana*), Rubiaceae (*Oxyanthus recemosus*), Caesalpiniaceae (*Cynometra megalofila*), Fabaceae (*Dialium guinnense*), Loganiaceae (*Strychnos* sp), Linaceae (*Hugonia planchonii*), and Annonaceae (*Xylopia parvifolia*).

A forest island (FI; 8°45′30.3″ N and 3°47′18.1″ W): a semi-deciduous forest island completely surrounded by savanna habitats. The tree density within this habitat is relatively low (1 to 3 large trees/100 m^2^), with a discontinuous tree canopy. The undergrowth of this habitat is dominated by lianas species and several new growth plants. The leaf litter on the soil is also less important in comparison to the gallery forest while a great quantity of dead woods with several levels of decomposition were observed. Borders of this habitat are subjected to annual bushfires. A total of 25 woody plant species were observed with the dominant species belonging to four families: Rubiaceae (*Oxyanthus racemosus*), Cesalpinaceae (*Dialium guineense*, *Cynometra megalophylla*), Apocynaceae (*Saba comorensis*), and Celastraceae (*Salacia* sp., *Salacia baumannii*).

A woodland savanna (WS; 8°46′04.9″ N and 3°47′31.1″ W): an extremely heterogeneous vegetation type consisting of a mixture of grass, shrub, and tree layers with a high rate of tree cover (>60%), as described by [22]. This habitat is also characterized by a discontinuous presence of leaf litter and a relatively poor abundance of fallen branches on the soil and is rarely completely burned. A total of 38 woody plant species belonging to eight major families were recorded: Annonaceae (*Uvaria chamoe*), Vitaceae (*Cissus populnea*), Rubiaceae (*Clausena anisata*), Sapotaceae (*Manikara multinervis*), Cesalpiniaceae (*Piliostigma thonningii*), Sapindaceae (*Allophylus africanus*), Combretaceae (*Anogeissus leiocarpus*), and Euphorbiaceae (*Antidesma venosum*).

A tree savanna (TS; 8°45′23.0″ N and 3°47′15.9″ W): an annually burned habitat with a woody plant species cover ranging from 7% to 36% [22] and a dominant herbaceous layer which forms the fuel of the annual bushfire. No leaf litter and fallen branches were observed on the soil. The impact of the annual fire is easily observable on the trunks of the woody plant species. Dominant plant species of this habitat belong to five families: Rubiaceae (*Crossopteryx febrifuga*), Combretaceae (*Terminalia macroptera*), Cesalpiniaceae (*Burkea africana*), Papilionceae (*Pericopsis laxiflora*), *Detarium microcarpum*, *Terminalia laxiflora*), and Poaceae (*Andropogon* sp. and *Hyparrheia* sp.).

A grassy savanna (GS; 8°46′14.6″ N and 3°47′06.1″ W): a homogenous fuel-rich extended herbaceous layer dominated by Poaceae (*Andropogon Africanus*, *Brachiara jubata*, *Echinochloa crusgalli*, *Ergrostis pilosa*, *Haparrheia* sp. and *Loudetia* sp.), with very rare trees and shrubs located at a distance from each other and often regrouped in groves consisting of *Crossopteryx lecardi* (Combretaceae) and *Entadas abyssinica* (Cesalpinaceae) species. This habitat is completely burned during the dry season.

Based on personal observations of fuel availability (i.e., herbaceous layer) and the size of the burned areas in each habitat during the previous years, the occurrence and impact of annual bushfire within these chosen habitats were determined through a range of values from 0 to 3; and assigned to habitats from the lowest to the highest occurrence, respectively, and impact of annual fire (Gallery Forest (GF) and Forest Island (FI) = 0; Woodland Savanna (WS) = 1; Tree Savanna (TS) = 2 and Grassy Savanna (GS) = 3). 

### 2.2. Sampling Design

#### 2.2.1. Assessing Termite Diversity and Abundance

Termites’ sampling was carried out in 2016, using a standardized belt transect protocol, first developed for sampling termites in forests [23] and then adapted to savannas [16,24]. In short, the protocol consists of a thorough search of dead plant material on the ground, on and in trees and mounds, as well as soil sampling to assess termite diversity [23]. Corresponding to the established protocols, in the savanna, each transect measures 50 m × 2 m, divided into ten 5 m × 2 m sections, while those for the forest are 100 m × 2 m with twenty 5 m × 2 m sections. Furthermore, while for the forest, three transects are established and sampled [23], five transects are sampled in savanna habitats [16]. Each transect section was systematically searched by a trained person for termites for 15 min in the savanna and 30 min in the forest. Additionally, eight soil scrapes per transect section measuring 15 cm × 15 cm × 10 cm have been sampled. Five transects have been sampled per visited habitat. All encountered termites were collected and stored in 96.5% ethanol for subsequent identification in the laboratory.

#### 2.2.2. Environmental Variables Characterization

In each habitat, three 50 m × 50 m plots were being established. Woody plant species richness and species abundance were determined in three subplots of 10 m × 10 m, arbitrarily located within each of these plots. Soil characteristics were determined by collecting samples with a ground auger at three layers of 20 cm (0–20, 20–40 and 40–60) at nine points in each habitat for future laboratory analyses [25]. Samples have been collected in the middle of the arbitrarily chosen subplots. All nine samples of each layer obtained in a habitat were mixed to get a composite sample (for details refer to [25]).

The correlation between a set of environmental variables and termite assemblages’ characteristics within the visited habitats was tested (i.e., habitat type, occurrence of annual bushfire, woody plant density, woody plant species richness, herbaceous species diversity, and soil physicochemical parameters (pH, organic carbon, nitrogen, clay, silt, sand…)).

#### 2.2.3. Identification of Specimens

Termite’s specimens have been morphologically identified at the morpho-species level using a low-power stereo microscope with a reticle (Leica MZ6), using standard identification keys [26,27,28,29] and region-specific illustrations and keys [30,31,32,33].

### 2.3. Data Analysis

The assessment of the sampling efficiency of termites during this study was tested by constructing sample-based species accumulation curves and by recording the mean similarity between transects of the same habitat type. These accumulations curves represent the evolution species number (species richness) according to the sampling effort (expressed in sampled sections). Using the program EstimateS 8.0.0 (Robert K. Colwell, Department of Ecology & Evolutionary Biology, University of Connecticut, Storrs, CT 06869-3043, USA; Website: http://purl.oclc.org/estimates), the observed and estimated species accumulation curves, respectively Sobs and Chao2, were constructed after randomizing the sample order 500 times to ensure the statistical representation of the target assemblage [34].

The second order and non-parametric estimator Chao2 was chosen as an estimator of the species richness, as suggested by [35] for incidence data. This parameter takes into account the distribution of species among sections and only needs, for its calculation, the number of species found in just one section and the number of species in exactly two. 

The total species richness corresponds to the total number of observed termite species in each habitat [36]. It was obtained by enumerating all species observed over the transects combined. The mean species richness represented the mean number of species found in the five transects of the same habitat. As sampling was based on the occurrence of individuals (presence–absence) rather than their numbers with respect to the social habit of termites, the relative abundance was considered as the number of encounters per transect, in which the presence of one species in a section represented one encounter [37]. 

The diversity of termite assemblages is measured by Simpson’s index. This index and its evenness were computed using the program, Ecological Methodology (Charles J. Krebs, University of British Columbia; Website: www. Zoology.ubc.ca/Krebs). To find out how widely assemblage compositions in habitat types were similar, cluster analysis in STATISTICA version 8.0.0 (www. statsoft.com) was used to assess similarities between them on the basis of the termite’s species. The matrix of dissimilarity was constructed using the complementarity or turnover values (i.e., rate of change in species identity = 1—Jaccard index of similarity) between habitats. An Unweighted Pair Group Method Using Arithmetic Averages (UPGMA) was used as the clustering method. The assessment of Jaccard’s similarity index of termite assemblages allowed for the description of the similarity between habitat types in terms of the species identity they harbor to be made [38]. The auto-similarity is defined as the mean similarity between the replicate transects of the same habitat [34]. Its calculation requires the two binary similarity indexes (Jaccard’s and Sorensen’s indexes) that could be determined using the program, EstimateS 8.0.0. As suggested by [16], ‘‘dominant’’ species were those whose total number of occurrences (from all habitats) equaled or exceeded the mean number of potential occurrences per habitat. 

Variations of feeding group abundances across habitat types were tested using the one-way analysis of variance whereas the least significant difference (LSD) post hoc comparison tests were performed to detect differences between species richness. 

The Canonical Correspondence Analysis (CCA) was used to investigate the effects of environmental variables on termite species’ occurrence and abundance [39] using the Ade4 package in R according to [40,41,42,43,44]. The aim of such a canonical ordination is to detect the main pattern in the relation between the termite species and the observed environmental variables [45]. For the clarity of the ordination and because of a strong collinearity between some variables, we first made a detailed examination of the correlation matrix of the environmental variables in order to identify the subset to be used for the correlation analyses by removing one of the highly correlated variables (*p* < 0.05) from the relative composition data of the CCA. The correlation matrix test was performed with the software R using the R-commander (Cmdr) package.

## 3. Results

### 3.1. Overall Taxonomic Structure of the Observed Species

Sixty species of termites, belonging to 19 genera, seven subfamilies and two families, namely Rhinotermitidae (Coptotermitinae and Rhinotermitinae) and Termitidae (Apicotermitinae, Cubitermitinae, Macrotermitinae, Nasutitermitinae, and Termitinae), were collected within all the visited habitats, see Appendix A. The highest number of species was observed in the Termitinae subfamily (20 species). Eighteen species belonging to the Macrotermitinae subfamily were recorded while eight species were collected both in the Cubitermitinae and Nasutitermitinae subfamilies. The less-representative subfamilies were the Apicotermitinae (three species), Coptotermitinae (two species) and Rhinotermitinae (one species). The collected species were assigned to the four feeding groups of termites: fungus growers (18 species), the wood-feeders (17 species), the soil-feeders (19 species), and the grass-feeders (6 species).

### 3.2. Sampling Efficiency during this Study

The mean of autosimilarity between transects was found to vary from one habitat to another. A small variation was observed between transects of both forests on one hand and those of the three savanna types on the other hand. The highest values of autosimilarity between transects were found in the grassy savanna (0.69) and the woodland savanna (0.52), suggesting a low difference of transects established in these habitats in terms of the specific composition of termites, see Table 1. In contrast, differences were observed in specific compositions between transects of the forest habitats and the shrubby savanna, as suggested by the calculation of the respective autosimilarity values (i.e., 0.43 in the forest gallery, 0.43 in the forest island, and 0.44 in the tree savanna). The highest number of unique species were recorded, respectively, in the gallery forest (10), woodland savanna (8) and tree savanna (7).

With five transects established in each of the visited habitats, a good picture of the community composition was obtained, see Table 1. The values of the sampling coverage showed that at least 85% of the expected species were sampled in each habitat (mean = 91.38%; Table 1). The highest sampling coverage was obtained in the forest island (95.8%) while the lowest one was noted in the gallery forest (85.33%). 

**Figure 1:**
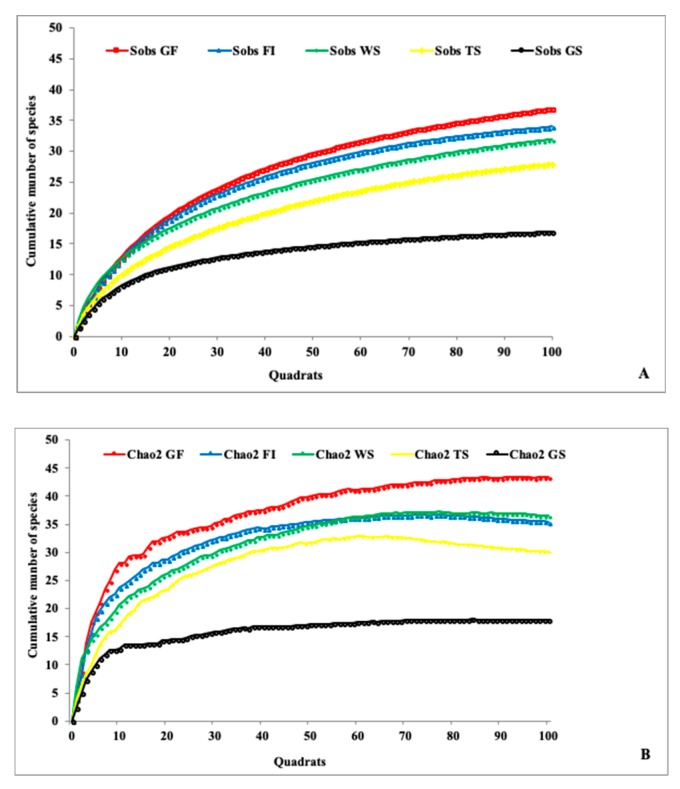
Accumulation curves of the observed (**A**) and estimated species richness (**B**) in the visited habitats.

The curves of the observed species accumulation and estimated species richness were similar in all the visited habitats, see Figure 1, implying that the species richness observed is a good estimate of that to be expected in the visited habitats.

### 3.3. Species Richness, Species Diversity, and Spatial Distribution of the Recorded Termites

A high value of Simpson’s diversity index was not found in all of the visited habitats, see Table 1. However, the highest values of this index were observed in the woody (0.56) and the grassy (0.51) savanna, while the lowest one was recorded in the forest island (0.46). Intermediate values of Simpson’s diversity index were obtained for the gallery forest (0.48) and the shrubby savanna (0.44). In contrast, the evenness presents high values which varied significantly from one habitat to another (χ^2^ = 43.58, ddl = 4, *p* < 0.01). The highest evenness was observed in the grassy savanna (0.89) and the smallest in the savanna woodland (0.66). However, when fixing the threshold of dissimilarity to 60% in this study, the results reveal that the forest habitats and the woodland savanna are similar, as shown in Table 2.

**Figure 2:**
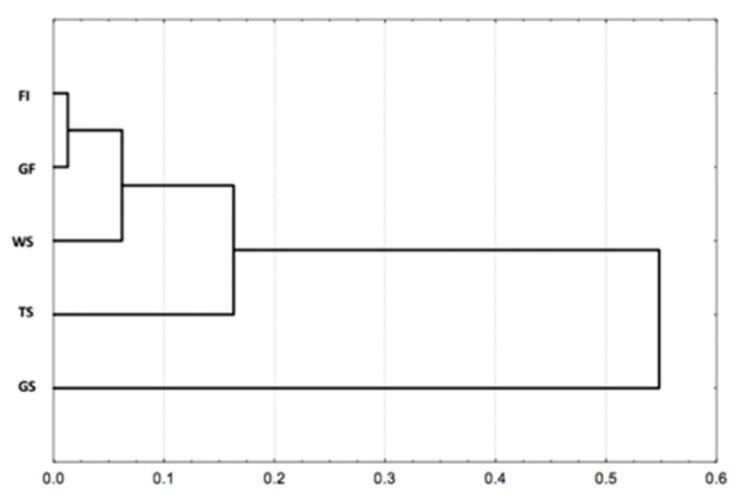
Classification of visited habitat types based on their respective termite’s species composition using the nearest-neighbor method (Unweighted Pair-Group Method using Arithmetic Averages), with 1—Jaccard index as the distance between groups. Abbreviations: FI = Forest Island, GF = Gallery Forest, WS = Woodland Savanna, TS = Tree Savanna, GS = Grassy Savanna.

A significant difference between the species richness of the visited habitats was also observed (χ^2^ = 46.97, ddl = 4, *p* < 0.001). This is evident in Table 1, from the large gap between the richest habitat (gallery forest with 37 recorded species) and the poorest habitat (grassy savanna with 17 species). Furthermore, the Hierarchical Ascending Classification (HAC), based on the species composition of the visited habitats, showed only one group even if forest habitats and the savanna woodland were similar and relatively different to the grassy savanna, see Figure 2. 

### 3.4. Relative Abundance of Termite’s Feeding Groups across the Visited Habitats

The four collected feeding groups of termites were encountered (i.e., fungus growers, soil feeders, wood feeders, and grass feeders). The abundance of these feeding groups was found to be significantly different from one habitat to another, except in the wood feeders’ group, see Table 3. Specifically, forest habitats were characterized by a total absence of grass feeders and the highest abundance of fungus growers (more than 50% of the recorded species in both forest habitats). However, all the feeding groups were recorded in the savanna habitats. Fungus growers remain the most dominant feeding group whatever the considered savanna habitat type. The highest relative abundance was found in fungus-growing termites within the woodland savanna, with 16 recorded species and a relative abundance of 64.68%, see Table 3. 

### 3.5. Effects of Environmental Variables on Species Distribution

The distribution of termite species was found to be correlated with habitat types and their respective environmental variables. The four axes of the Canonical Correspondence Analysis (CCA) explained 100% of the ordination of habitat-specific diversity of the recorded termites, dependent on environmental variables, see Table 4. This distribution was found to be mainly determined by the first two axes (i.e., 69.58%). Both axes, formed by the Fire Occurrence (FO), Woody Plant Diversity (WPD), Organic Carbon (OC), and Herbaceous Species Richness (HSR), mainly explained the distribution of termites’ species within the visited habitats. The Fire Occurrence (FO), Herbaceous Species Richness (HSR), and the pH were found to be highly correlated with the distribution of species of the genus, *Trinervitermes*. In contrast, the distribution of forest-dwelling species (*Macrotermes* spp., *Cubitermes* spp.) was found to be highly correlated with Woody Plant Diversity (WPD), Woody Plant Families Diversity (WPFD), and Organic Carbon (OC).

**Figure 3:**
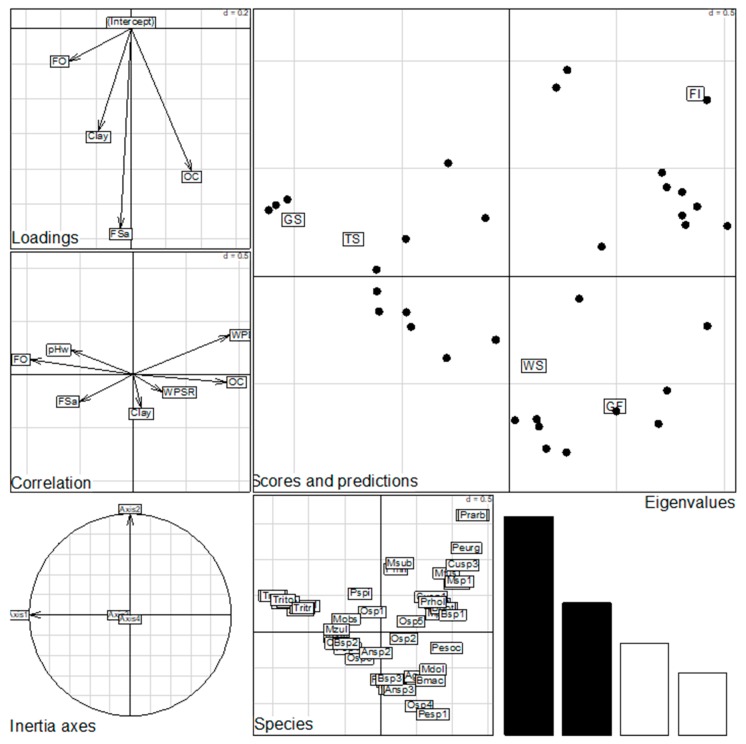
Diagram of CCA ordination of habitat-specific diversity and abundance of collected termites in dependence of environmental variables.

**Figure 4:**
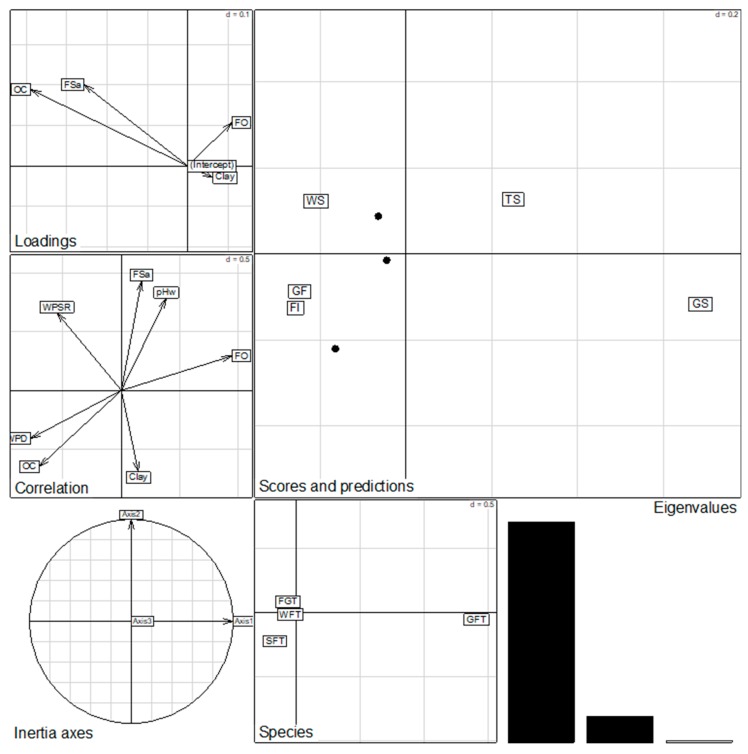
Diagram of CCA ordination of habitat-specific abundance of collected termite’s feeding groups in dependence of environmental variables.

The distribution of feeding groups (i.e., diversity and abundance of each group) was also found to be mainly controlled by the habitat types in general and, specifically, by a set of environmental variables, see Figure 3. Grass feeders were encountered only in tree and grassy savannas while soil and wood feeders were collected in forest habitats (gallery and island forests). The fungus-growing termites were mainly found distributed in the savanna woodland. The Canonical Correspondence Analysis (CCA) showed that this distribution is mainly determined by the FO and the HSR for grass feeders and by the Woody Plant Species Richness (WPSR) for wood feeders and fungus-growing termites, see Figure 4. In contrast, the distribution of soil feeders was found to be highly correlated with the Woody Plant Diversity (WPD).

## 4. Discussion

Sixty species of termites (e.g., good sampling coverage with more than 85% of the expected termites’ species of each visited habitat) were recorded during this study within all the visited habitats. This diversity is higher in comparison to the 31 observed species of the same habitat types within a Guinean savanna of the Lamto Reserve in Central Côte d’Ivoire [16]. This observation is probably due to (i) the highest floristic diversity (e.g., diversified food sources for termites) of the Sudano-Guinean savanna of the Comoe National Park and (ii) the relative stability of the ecosystems of this park in terms of anthropic activities. However, the highest values of the species richness were found in forest habitats (i.e., 37 species in the gallery forest and 34 species in the forest island). Moreover, relatively high species richness was also found in savanna habitats (32 species in woodland savanna and 28 species in tree savanna), with, of course, a low complementarity between termites’ assemblages of forests and savanna habitats. The highest species richness observed in the forest habitats was suggested to be due to the high stability of these ecosystems in comparison to savannas which are annually burnt [14,46,47,48]. The lowest species richness observed in the grassy savanna could mainly be explained by this annual disturbance. Fire behavior (intensity, the rate of spread, flame height, residence time, and surface temperature) is influenced by a wide range of variables such as fuel characteristics, burning season, and weather conditions [49,50,51,52]. Grassy savanna is known to be completely burnt each year, leading to a relatively high instability of this habitat type, with a less diversified food availability.

The abundance of the termite feeding groups was found to vary significantly from one habitat to another. Fungus-growing termites, soil feeders, and wood feeders were observed in all the visited habitats while grass feeders were found only in the savanna ecosystems. In addition, the highest occurrence has been observed in the fungus growers, followed by the wood, soil and grass feeders, respectively. This feeding group was judged by several authors [25,53,54] not to be strongly affected by habitat variability. These termites are characterized by their high ecological plasticity based more on the adaptability of their nests across habitats than their own physiological adaptability and their symbiotic relationship with fungi of the genus *Termitomyces*, allowing them to feed on a wide range of plant species litter [55]. However, a recent study [56] revealed a potential negative effect of disturbances on these organism’s, contrary to what has been said in the literature. The authors of that study proposed the use of a combination of both methods (Rapid Assessment Protocol and the TSBF monoliths) in order to test the ambiguous behavior of fungus-growing termites in response to anthropogenic pressure. The specificity of the visited habitat type and the food preference of each feeding group can also, for the most part, explain the observed results. Indeed, forest habitats are characterized by an exclusive presence of trees while savannas are defined as mixed tree-grass communities, comprising systems with a continuous herbaceous layer and a discontinuous woody stratum [57]. The exclusive presence of grass feeders in these savanna habitats shows the high importance of food availability and specificity with regard to the species that occur within habitats. For example, the woodland savanna, which is an intermediate habitat between forest and savanna (i.e., presence of grass and a relatively high density of trees) with very little annual bushfire disturbance, was found to be relatively similar to forest habitats (Hierarchical Ascending Classification). This observation is also confirmed by the highest total occurrence of soil feeders within forest habitats due to the high quantity and quality of leaf litter, humus, twigs, and fallen branches in these habitats. This is in agreement with [58], which showed the correlation between the abundance of soil feeders of a habitat and the high rate of its organic matter. 

The distribution and abundance of termites were found to be significantly correlated with a set of environmental variables such as Fire Occurrence (FO), Herbaceous Species Richness (HSR), Woody Plant Species Richness (WPSR), Woody Plant Diversity (WPD), Woody plant Families Diversity (WPFD), and Organic Carbon (OC). However, Fire Occurrence (FO) seems to be the most important disturbance of the study sites, modeling termite assemblage’s characteristics within habitats. The FO appears to indirectly impact the diversity, relative abundance, and distribution of termites within the visited habitats [59]. Habitats which are not annually burnt and are, therefore, supposed to be more stable, were found to host the highest diversity and abundance of termites. Termites of the grass feeders’ group were exclusively recorded in these so-called disturbed habitats (i.e., savanna habitats annually burnt) suggesting these termites are not sensitive to the annual bushfires. Indeed, as suggested by [59], termites respond differently and opposingly to burning. In general, they are highly resistant to this annual disturbance in wet savanna. Some feeding groups (i.e., wood and grass feeders) are identified as highly resistant to fire while soil feeders were rarely found on burnt habitats.

## 5. Conclusions

This study has provided new information regarding the effects of a set of biotic and abiotic factors of habitats on the diversity and distribution of termite communities. The structure of these communities varies according to habitat type and their respective environmental parameters. The highest species richness was found in forest habitats; while a relatively good species richness was also observed in savanna habitats, with, of course, a low complementarity between termites’ assemblages within forests and savanna habitats. The visited Sudano-Guinean savanna ecosystem was considered to be unstable, and when modeled by the uncontrolled annual bushfire and herbivory was characterized by an important spatial heterogeneity. This offers a diversified natural habitat with an important variety of termite-habitat specific species (i.e., diversified and high plant diversity leading to a good availability of food). The maintenance of such a termite assemblage’s diversity is determined by the proper and sustainable management of annual bushfire and the long-lasting conservation of herbivore’s populations of this park. Indeed, without these disturbances, trees are expected to outcompete grass and change this useful spatial heterogeneity, which was responsible for the great species richness of termites that was observed.

## Figures and Tables

**Table 1:** Metrics of termite diversity in the different habitats.

Habitat Types	Total Species Richness	Sampled Coverage	Similarity between Transects	Simpson’s Index	Evenness’s Index	Abundance	Unique Species per Habitat
Gallery Forest	37	85.33	0.43	0.48	0.78	209	10
Forest Island	34	95.8	0.43	0.46	0.84	187	6
Woodland Savanna	32	87.77	0.52	0.56	0.66	252	8
Tree Savanna	28	93.09	0.44	0.48	0.81	209	7
Grassy Savanna	17	94.5	0.69	0.51	0.89	141	3

**Table 2:** Dissimilarity assessment of termite assemblages between the visited habitats.

Habitat Types	GF	FI	WS	TS	GS
Gallery Forest	0				
Forest Island	22/27/55%	0			
Woodland Savanna	20/29/59%	23/20/50%	0		
Tree Savanna	17/31/**65%**	15/32/**68%**	21/19/46%	0	
Grassy Savanna	10/34/**77%**	9/33/**79%**	11/27/**71%**	14/17/54%	0

Data in cells are reported as follows: common species/different species/1-Jaccard’s similarity index (%). Scores in bold indicate high dissimilarity.

**Table 3:** Variation in relative abundances of termite feeding groups within the visited habitats.

Habitats							
Feeding Groups	GF	FI	WS	TS	GS	Total	*p* Values
Fungus growers	113 (54.06)	99 (52.94)	157 (64.68)	130 (62.2)	61 (42.95)	560 (56.05)	0.001
Wood feeders	40 (19.13)	43 (23)	52 (19.33)	41 (19.61)	27 (19.01)	203 (20.32)	0.156
Soil feeders	56 (26.8)	45 (24.06)	42 (15.61)	28 (13.39)	24 (16.9)	195 (19.51)	0.014
Grass feeders	0	0	1 (0.37)	10 (4.78)	30 (21.12)	41 (4.1)	0.001
*p* Values	<0.001	<0.001	<0.001	<0.001	<0.001	-	-

Abbreviations: GF = Gallery Forest; FI = Forest Island; WS = Woodland Savanna; TS = Tree Savanna and GS = Grassy Savanna. Values in brackets represent the percentage of each feeding group within every visited habitat.

**Table 4:** Summary statistic for the Canonical Correspondence Analysis (CCA) ordination of termites’ species and feeding groups diversities and abundance as a dependence of environmental variables.

Summary Statistic for CCA Ordination	Habitat-Specific Diversity and Abundance of Collected Termites in Dependence of Environmental Variables				Habitat-Specific Diversity of Collected Termite’s Feeding Groups in Dependence of Environmental Variables				Habitat-Specific Abundance of Collected Termite’s Feeding Groups in Dependence of Environmental Variables			
Axes	1	2	3	4	1	2	3	4	1	2	3	4
Eigenvalues	0.45	0.27	0.19	0.13	0.15	0.02	0	-	0.11	0.01	0	-
Projected inertia (%)	43.35	26.24	18.13	12.29	84.74	12.05	3.2	-	88.6	10.57	0.82	-
Cumulative projected inertia (%)	43.35	69.58	87.71	100	84.74	96.79	100	-	88.61	99.18	100	-
Total unconstrained inertia	0.10				0.18				0.13			
Inertia (%)	100				100				100			

## Appendix A

**Table A1:** List of species and morphospecies recorded in the visited habitats of the Comoe National Park.

Termites Species (Feeding Group/Family/Subfamily)	Habitats				
	FI	GF	WS	TS	GS
**Fungus growers—Termitidae—Macrotermitinae**					
*Ancistrotermes cavithorax* (Sjöstedt, 1899)	2	15	39	62	29
*Ancistrotermes crucifer* (Sjöstedt, 1897)	26	10	19	0	0
*Ancistrotermes guineensis* (Silvestri, 1912)	14	45	56	1	1
*Macrotermes bellicosus* (Smeathman, 1781)	7	4	2	1	0
*Macrotermes subhyalinus* (Rambur, 1842)	12	0	2	5	3
*Microtermes osborni* (Emerson, 1928)	8	3	16	30	10
*Microtermes toumodiensis* (Grassé, 1937)	0	4	0	0	0
*Pseudacanthotermes militaris* (Hagen, 1858)	11	0	2	9	0
*Pseudacanthotermes spiniger* (Sjöstedt, 1900)	4	0	2	9	0
*Pseudacanthotermes* sp1	0	0	2	2	0
*Odontotermes* sp1	10	9	4	2	15
*Odontotermes* sp2	3	5	2	2	1
*Odontotermes* sp3	0	8	4	4	0
*Odontotermes* sp4	0	4	2	0	0
*Odontotermes* sp5	2	1	2	1	0
*Odontotermes* sp6	0	3	2	2	2
*Odontotermes* sp7	0	2	0	0	0
*Odontotermes* sp8	0	0	1	0	0
**Wood feeders—Rhinotermitidae—Coptotermitinae**					
*Coptotermes intermedius* (Sylvestri, 1912)	0	6	0	0	0
*Coptotermes sjoestedti* (Harris, 1966)	0	2	0	0	3
**Wood feeders—Termitidae—Nasutitermitinae**					
*Fulleritermes tenebricus* (Silvestri, 1914)	0	0	23	3	0
*Nasutitermes arborum* (Smeathman, 1781)	13	6	1	0	0
**Wood feeders—Rhinotermitidae—Rhinotermitinae**					
*Shedorhinotermes laminianus* (Sjöstedt, 1911)	2	0	2	0	0
**Wood feeders—Termitidae—Termitinae**					
*Amitermes evuncifer* (Silvestri, 1912)	0	1	0	13	6
*Amitermes lonnbergianus* (Sjöstedt, 1911)	0	1	0	0	0
*Amitermes* sp	0	0	1	0	0
*Microcerotermes choanensis* (Silvestri, 1914)	1	0	0	0	0
*Microcerotermes dolichognathus* (Sjöstedt, 1926)	1	3	1	0	0
*Microcerotermes fuscotibialis* (Sjöstedt, 1896)	2	0	1	0	0
*Microcerotermes parvulus* (Sjöstedt, 1911)	2	1	1	0	0
*Microcerotermes parvus* (Haviland, 1898)	17	11	8	1	0
*Microcerotermes zuluensis* (Sjöstedt, 1926)	3	7	14	24	17
*Microcerotermes* sp1	2	1	0	0	0
*Microcerotermes* sp2	0	0	0	0	1
*Termes* sp1	0	1	0	0	0
**Soil feeders—Termitidae—Apicotermitinae**					
*Adaiphrotermes* sp	1	0	2	10	9
*Aderitotermes* sp	7	21	28	11	15
*Astratotermes* sp	0	7	6	2	0
**Soil feeders—Termitidae—Cubitermitinae**					
*Basidentitermes potens* (Silvestri, 1914)	2	1	1	0	0
*Basidentitermes mactus* (Silvestri, 1914)	2	9	3	0	0
*Basidentitermes* sp1	1	1	0	0	0
*Basidentitermes* sp2	0	1	0	2	0
*Basidentitermes* sp3	0	4	0	2	0
*Cubitermes* sp1	3	2	0	1	0
*Cubitermes* sp2	3	0	0	0	0
*Cubitermes* sp3	6	2	0	0	0
**Soil feeders—Termitidae—Termitinae**					
*Pericapritermes appelans* (Silvestri, 1914)	0	2	0	0	0
*Pericapritermes silvestrianus* (Emerson, 1928)	1	0	0	0	0
*Pericapritermes socialis* (Sjöstedt 1965)	1	2	0	0	0
*Pericapritermes urgens* (Silvestri 1914)	5	1	0	0	0
*Pericapritermes* sp1	0	3	0	0	0
*Procubitermes aburiensis* (Sjöstedt, 1926)	5	0	0	0	0
*Promirotermes holmgreni* (Silvestri, 1912)	2	0	2	0	0
*Procubitermes sjoestedti* (Rosen, 1912)	6	0	0	0	0
**Grass feeders—Termitidae—Nasutitermitinae**					
*Trinervitermes geminatus* (Wasmann, 1897)	0	0	1	1	12
*Trinervitermes occidentalis* (Sjöstedt, 1904)	0	0	0	0	11
*Trinervitermes oeconomus* (Trägårdh, 1904)	0	0	0	4	0
*Trinervitermes togoensis* (Sjöstedt, 1899)	0	0	0	2	4
*Trinervitermes rhodesiensis* (Sjöstedt, 1911)	0	0	0	2	2
*Trinervitermes* sp1	0	0	0	1	0

Abbreviations: FI: Forest Island, GF: Gallery Forest, WS: Woodland Savanna, TS: Tree Savanna, GS: Grassy Savanna. Scores represent the frequency of occurrence of every species within the visited habitats.
